# A framework for large-scale mapping of human settlement extent from Sentinel-2 images via fully convolutional neural networks

**DOI:** 10.1016/j.isprsjprs.2020.01.028

**Published:** 2020-05

**Authors:** Chunping Qiu, Michael Schmitt, Christian Geiß, Tzu-Hsin Karen Chen, Xiao Xiang Zhu

**Affiliations:** a Signal Processing in Earth Observation (SiPEO), Technical University of Munich (TUM), Arcisstr. 21, 80333 Munich, Germany; b German Remote Sensing Data Center (DFD), German Aerospace Center (DLR), Oberpfaffenhofen, 82234 Wessling, Germany; c Department of Environmental Science, Aarhus University, Frederiksborgvej 399, DK-4000 Roskilde, Denmark; d Remote Sensing Technology Institute (IMF), German Aerospace Center (DLR), Oberpfaffenhofen, 82234 Wessling, Germany

**Keywords:** Built-up area, Convolutional neural networks, Human settlement extent, Sentinel-2, Urbanization

## Abstract

Human settlement extent (HSE) information is a valuable indicator of world-wide urbanization as well as the resulting human pressure on the natural environment. Therefore, mapping HSE is critical for various environmental issues at local, regional, and even global scales. This paper presents a deep-learning-based framework to automatically map HSE from multi-spectral Sentinel-2 data using regionally available geo-products as training labels. A straightforward, simple, yet effective fully convolutional network-based architecture, Sen2HSE, is implemented as an example for semantic segmentation within the framework. The framework is validated against both manually labelled checking points distributed evenly over the test areas, and the OpenStreetMap building layer. The HSE mapping results were extensively compared to several baseline products in order to thoroughly evaluate the effectiveness of the proposed HSE mapping framework. The HSE mapping power is consistently demonstrated over 10 representative areas across the world. We also present one regional-scale and one country-wide HSE mapping example from our framework to show the potential for upscaling. The results of this study contribute to the generalization of the applicability of CNN-based approaches for large-scale urban mapping to cases where no up-to-date and accurate ground truth is available, as well as the subsequent monitor of global urbanization.

## 1 Introduction

Human settlement extent (HSE), which is characterized by buildings, roads, and other man-made structures, is an essential indicator of the human footprint on the Earth. Moreover, it is an expression of the impact of ongoing worldwide urbanization. According to (United Nations, 2018), 55% of the world's population now lives in urban areas, a proportion that is expected to increase to 68% by 2050. To better understand drivers and interactions between urbanization and social and environmental processes, it is thus necessary to obtain accurate and up-to-date HSE data.

Recent years have seen a proliferation of studies related to HSE mapping, among which remote sensing-based approaches have gained more and more attention due to their inherent ability to frequently and regularly observe the land surface on a global scale. With this unique property, several remote sensing-based global products related to HSE have become available. One, the Global Urban Footprint (GUF), was derived using TerraSAR-X as well as TanDEM-X Synthetic Aperture Radar (SAR) images (Esch et al., 2012, Esch et al., 2013). Another, the Global Human Settlement (GHS) built-up grid, was derived from the Landsat as well as the Sentinel-1 image collections. GHS built-up grid is a product derived within the GHSL image analytics framework, which also utilizes remote sensing images from other missions such as SPOT-5 and 6 (Pesaresi et al., 1975, Corbane et al., 2017). Still others, the GlobeLand30 land cover map and the Global Human Built-up And Settlement Extent (HBASE), were derived from the 30 m resolution Landsat data (Chen et al., 2017, Wang et al., 2017). There are several other global land cover maps, such as finer resolution observation and monitoring of global land cover with 30 m (FROM-GLC30) and 10 m (FROM-GLC10) resolution, Global Land Cover 2000 (GLC2000) with 1 km resolution, and those derived from Moderate Resolution Imaging Spectrometer (MODIS) data with 500 m resolution, which are also produced using remote sensing image analysis (Gong et al., 2013, Gong et al., 2015, Bartholome and Belward, 2005, Friedl et al., 2002). It is difficult to compare these products directly as they each have slightly different foci. Generally, among these products, GUF outperforms the others (Marconcini et al., 2019), especially in rural areas where most of the products fail to detect impervious surfaces. GUF, however, is not feasible for frequent update as it was derived from the relatively expensive high resolution TerraSAR-X and TanDEM-X SAR images.

Novel approaches for urban mapping explore cloud computing services like Google Earth Engine and the large amount of remote sensing data it offers (Patel et al., 2015, Goldblatt et al., 2018, Liu et al., 2018). In these examples, it is expected that the globally available multi-spectral Sentinel-2 data, with a 5-day temporal resolution and 10-meter spatial resolution, are going to play a key role in more accurate HSE mapping at a large or even global scale, with the potential for frequent monitoring of global urbanization. This is already being shown by some regional-scale studies, with similar applications on urban impervious surface mapping (Xu et al., 2018) and land cover mapping (Gong et al., 2015, Qiu et al., 2019).

In the past, urban mapping approaches typically started by extracting hand-crafted features such as the normalized difference spectral vector (NDSV) and the gray-level co-occurrence matrix (GLCM), followed by feeding the extracted features into a traditional classifier such as Random Forests (Patel et al., 2015, Ban et al., 2015, Chini et al., 2018), and ending with post-processing to remove potential mis-classifications. However, as a form of semantic segmentation task (or pixel level labeling), HSE mapping can theoretically be carried out through deep learning-based approaches, because plenty of neural network architectures have been proposed and shown to be powerful for semantic segmentation tasks. For example, SegNet, U-Net, the deconvolution network, as well as other improved variants based on multi-scale context fusion, attention mechanisms, and recurrent neural networks, were all proposed after fully convolutional networks (FCNs) were introduced in 2015 (Badrinarayanan et al., 2017, Long et al., 2015, Noh et al., 2015, Badrinarayanan et al., 2017, Ronneberger et al., 2015). The fundamental advantage of all these deep neural networks is their ability for enhanced feature representation and pixel-level recognition. Examples where convolutional neural networks (CNN) and, in particular, FCNs are used for remote sensing image classification or segmentation include (Paisitkriangkrai et al., 2016, Maggiori et al., 2016, Längkvist et al., 2016, Maggiori et al., 2016, Fu et al., 2017, Volpi and Tuia, 2016, Rußwurm and Körner, 2018, Zhang et al., 2019, Zhong et al., 2019, Hu et al., 2019, Lang et al., 2019, He et al., 2018). Apart from the works focusing on very high resolution satellite or aerial imagery (i.e., with a ground sampling distance equal to or even less than 1 m), data of lower spatial resolution is also being studied, since the images of lower resolutions such as globally openly available Sentinel-2 imagery remain the key candidates for large-scale mapping (Helber et al., 2019, Sumbul et al., 2019).

Good performance, however, is not guaranteed when directly employing these existing approaches for large-scale HSE mapping from Sentinel-2 images. There are three reasons for this, each with possible solutions. First, getting sufficient reliable pixel-wise ground truth data, a major prerequisite for deep learning-based approaches, is more challenging than labelling standard photos that are the main subject of computer vision research. Therefore, we suggest to create annotations by exploiting geo-referenced map products such as the CORINE Land Cover data (Sumbul et al., 2019) and the MOD500 data (Schmitt et al., 2019, He et al., 2018), as well as governmental data (Rußwurm and Körner, 2018), which contains information relevant to the task one seeks to achieve. Second, remote sensing images differ significantly in appearance from the close-range images used in the standard literature on scene segmentation (Zhu et al., 2017). As mentioned before, remote sensing images are usually not with the same high resolution, and multi-spectral remote sensing images come with more bands than conventional photographs. Furthermore, they usually capture large geographical areas with different kinds of land cover, with occlusions, and with illumination changing over time and space. Taking these characteristics into account, downsampling should be avoided to fully exploit the rich information within the remote sensing data. Finally, the specific application scenarios, which in this study is large-scale or even global HSE mapping, should always be taken into account in the whole framework. This means that a spatial split of training and test data should be well designed (Geiß et al., 2017), and it is not enough to train a model with high test accuracy on a single experimental test set. Instead, the framework should include further applying the trained model on images acquired over all potential regions of interest, for which reasonable accuracy should also be achieved. This requires a robust model in the face of spectral signature changes resulting from social and cultural differences and changing acquisition conditions. Therefore, an independent accuracy assessment should be carried out in order to comprehensively assess the mapping results. In this way, a reliable interpretation and understanding of the performance of the framework will be gained.

This paper will present a framework that takes into account the three problems described above, by fully exploiting state-of-the-art algorithms and techniques, as well as the freely available global satellite images of the Sentinel-2 mission for large-scale HSE mapping. We propose a framework for large-scale HSE mapping from Sentinel-2 imagery using deep learning-based approaches with three major parts: (1) preparation of labels and image data, (2) training a well-generalizing semantic segmentation network to learn to map HSE from Sentinel-2 images (Sen2HSE-Net), and (3) a statistically sound accuracy assessment of the HSE results. This study is intended to provide answers to the following questions: How can large-scale HSE mapping benefit from CNNs and remote sensing images of medium resolution, in a situation where potentially noisy ground truth data is only available at a regional scale? How will the network architecture and experimental setup affect the mapping results? How good are the resulting HSE maps, compared to the existing state-of-the-art products derived at a similar scale?

The remainder of this paper proceeds as follows: Section 2 elaborates the proposed HSE mapping approach. Section 3 details descriptions about the study area and the experimental setup. Section 4 evaluates the HSE mapping accuracy and visualizes and compares the produced HSE maps to GUF, the GHS built-up grid, and other datasets from recent studies such as FROM-GLC10, for several sample test scenes. The following Section 5 provides answers to the questions raised above, based on the interpretation and analysis of the achieved results, and discusses the remaining challenges and the possible solutions for the future work. Finally, Section 6 summarizes and concludes the work.

## 2 HSE mapping with Sen2HSE-Net

Considering the spatial resolution of available reference data (20 m), the sub-pixel geolocation accuracy of Sentinel-2 data (Drusch et al., 2012), as well as the resolution of existing related products (mostly lower than 20 m), the specific goal of HSE mapping in this study is to detect whether buildings, roads, or other man-made structures are presented—that is, larger than 0% in a 20×20 cell. Using this definition, the resulting HSE output from Sentinel-2 imagery will be a binary layer in the Universal Transverse Mercator (UTM) coordinate system, with a ground sampling distance (GSD) of 20 m. This definition is also consistent with the 30 m Global Human Built-up and Settlement Extent (HBASE) dataset derived from Landsat, which consists of human settlement, built-up areas, and roads (Wang et al., 2017).

**Fig. 1:**
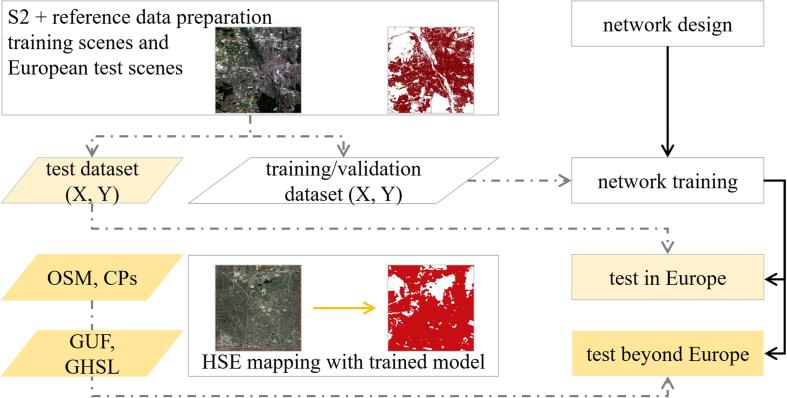
Generalized framework for HSE mapping. The network is instanced as Sen2HSE-Net and compared with several baselines in this study. X and Y are Sentinel-2 image patch and HSE label, respectively.

The procedure used in the proposed HSE mapping framework is illustrated in Fig. 1, which consists of image and reference data preparation, deep neural segmentation network training, and HSE mapping and assessment. Each step will be detailed in the following subsections.

### 2.1 Sentinel-2 image pre-processing and reference ground truth preparation

For each of the cities under study, one (mostly) cloud-free Sentinel-2 image is prepared with Google Earth Engine (GEE) (Gorelick et al., 2017), by exploring a cloud-based engineering approach. The processing approach, described in detail in (Schmitt et al., 2019), relies on pixel-wise cloud detection and the combination of multi-temporal images within short time periods. For each study area, we used three Sentinel-2 images compiled from all data acquired for spring, summer, and autumn 2017. The image data contains 13 spectral bands representing Top of Atmosphere Reflectance scaled by a factor of 10000. These images are orthoimages in UTM projection. We used ten of the bands: specifically, the channels with a GSD of 10, B2 (blue), B3 (green), B4 (red), and B8 (Near-infrared), as well as the 20 GSD bands, B5 (red edge 1), B6 (red edge 2), B7 (red edge 3), B8a (red edge 4), B11 (short-wavelength infrared 1), and B12 (short-wavelength infrared 2). In order to create composites with a consistent image size, we up-sampled the second group of bands to a GSD of 10 using cubic resampling. The employed reference data is “High Resolution Layer Imperviousness 2015,” an operational product, released as part of the Copernicus Land Monitoring Service's product portfolio (Langanke and Land, 2016). “High Resolution Layer Imperviousness 2015” is a raster layer indicating built-up areas with a spatial resolution of 20 m, created from Copernicus high resolution remote sensing images (mainly the Indian Remote Sensing Satellite and SPOT 5). It is produced using supervised classification, NDVI-based calibration, and subsequent visual improvement. The producer and user accuracies are supposed to be about 90%. For registration of reference data and Sentinel-2 images, the reference data is re-projected to the UTM coordinate system and resampled to the extent of the corresponding images.

**Fig. 2:**
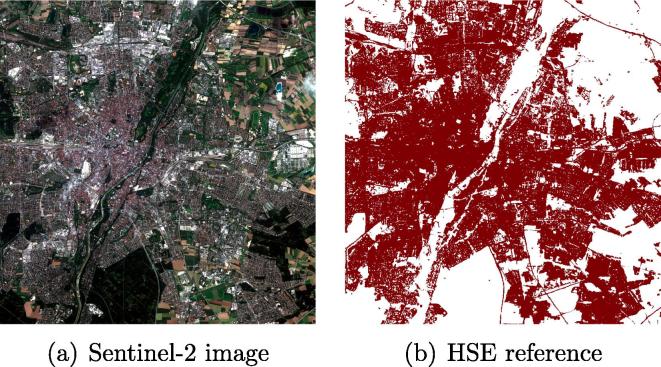
The processed Sentinel-2 image of central Munich, Germany, and the reference data.

As an example, Fig. 2 illustrates the processed Sentinel-2 image of central Munich, Germany, and the reference data.

### 2.2 Convolutional neural networks for semantic segmentation

CNNs currently are the state of the art in visual recognition tasks such as classification and detection, due to their ability to learn multi-scale representations with high predictive power from example data. They usually consist of basic layers such as convolutional layers composed of weights and biases, pooling layers for a summary of connected activations in feature maps, and activation layers for injecting non-linearity into the models. Some recent examples architectures include forms of the residual convolutional neural network (ResNet), ResNeXt, Inception, and Xception (He et al., 2016, Xie et al., 2017, Szegedy et al., 2015, Chollet, 2017), among many others. FCNs and their extensions inherit the basic structure of CNNs and replace the fully connected layer, i.e., the last layer in the CNNs, with a fully convolutional layer. They feature downsampling (encoder) together with subsequent upsampling (decoder) to maintain the resolution of the input image in the output map.

There are two approaches for remote sensing image classification via deep learning: working with either patch-based CNNs designed for image classification (Paisitkriangkrai et al., 2016, Längkvist et al., 2016, Rußwurm and Körner, 2018, Zhang et al., 2019, Zhong et al., 2019, Hua et al., 2019a, Hua et al., 2019b, Zhu et al., 2019) or encoder-decoder-like neural networks designed for semantic segmentation (Maggiori et al., 2016, Maggiori et al., 2016, Fu et al., 2017, Volpi and Tuia, 2016). The former works under the assumption of just a single label for each image patch, and applies the trained model to the image of a study area via a sliding window approach, with the target GSD as the stride of the sliding window. In contrast, the latter approach, FCNs are designed to predict pixel-level labels, and after training, they can accept inputs of arbitrary size. Their advantages are a potentially higher accuracy resulting from the inter-patch context information (only the intra-patch context is considered in patch-based CNN approaches), and less expensive computation, since overlapping patches are avoided when using the sliding window method for dense prediction.

Given both the goal of our task—to assign a label, HSE or non-HSE, to each 20×20 meter patch—and the advantages of pixel-level recognition, we decided to combine the patch-based CNN approach and pixel-level recognition approach. Instead of inputting a 20×20 meter patch into the network and outputting one label for the patch, we feed larger patches to the network and predict labels for each 2×2 pixels by including one pooling (downsampling) layer in the network.

### 2.3 Architecture and training of Sen2HSE-Net

**Fig. 3:**
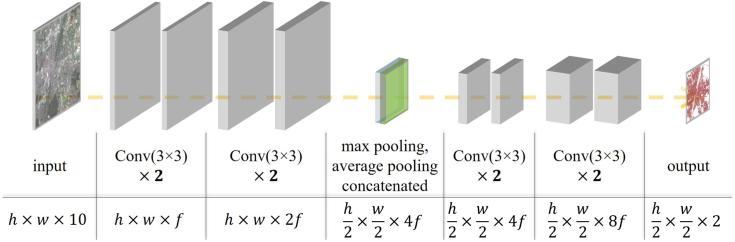
Architecture and details of Sen2HSE-Net. The terms “h”, “w”, and “f” denote height, width, and the channel number of the first feature maps, respectively. The different size of the final prediction from the input image is due to the different resolution of the HSE prediction (with a 20-meter GSD) to the input image (with a 10-meter GSD).

Considering that the network should be kept as simple as possible to make it feasible for reproduction and upscaling, we implemented a simple FCN, the architecture of which is illustrated in Fig. 3. It consists of four convolutional layers in the beginning to extract low-level features from the input Sentinel-2 images, two pooling layers (maximum and average pooling) in the middle to abstract the learned features to a higher level, then four convolutional layers to extract high-level features, and one convolutional layer in the end for predictions. The kernel sizes for the two sets of four convolutional layers are 3×3; the last convolutional layer has a kernel size of 1×1. Additionally, there are two drop-out layers to avoid model overfitting to the training data, given that the goal is to map HSE globally. No additional pooling layers are used to avoid the information loss during downsampling process, which is also the design idea in (Lang et al., 2019) and (Hasanpour et al., 2016). As defined, the output prediction is with a 20-meter GSD, while the input data is with a 10-meter GSD; thus no upsampling layers are used.

Filter weights are initialized using the algorithm proposed by (He et al., 2015). The number of output filters of the first convolutional layer, f, is set as 16 in the experiments and adjusted for investigations in Section 5. The input images and their corresponding reference labels are used to train the network with the Nesterov Adam optimizer implementation of Keras (Chollet, et al., 2015). We used a minibatch size of 8 images and fixed learning rate of $2\times 10^{-4}$. To control the training time and avoid overfitting, early stopping was used, and the monitored metric is the validation loss with patience of 10 epochs, which means that the training stops if the validation loss does not decrease for 10 epochs. All the experiments were carried out using the same setups described above, in order to make for meaningful comparisons.

## 3 Experimental setup

### 3.1 Study area and training data preparation

**Fig. 4:**
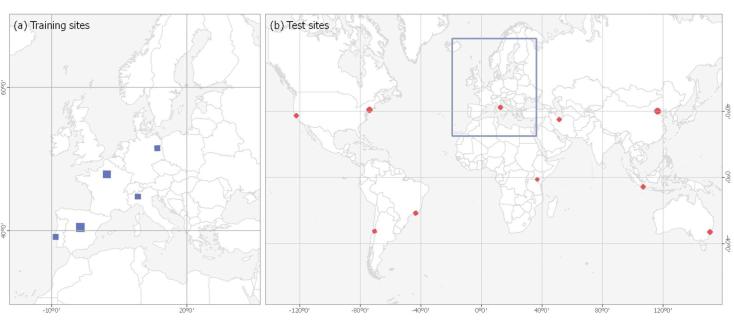
Five training areas distributed across Europe and ten test areas across the world.

The training areas are five cities in Central Europe, as shown in Fig. 4. These cities are chosen for training because the reference ground truth data is only available in Europe. The test areas are ten cities across the world, as shown in Fig. 4. In addition to these ten test scenes distributed across the world, three test scenes in Europe are also chosen to provide a basis for evaluating the regional-to-global generalization capability of the proposed framework. Table 1 describes the main characteristics of the selected test cities, which differ in urban area, topography, and land-cover features in the surrounding countryside.

**Table 1:** Basic information of the study areas for training and test, and the urban ecoregions according to (Schneider et al., 2010).

	City	Urban ecoregion	Area ($km^{2}$)
Training scenes	Berlin, Germany	Temperate forest in Europe	5138
	Lisbon, Portugal	Temperate mediterranean	4585
	Madrid, Spain	Temperate mediterranean	19,360
	Milan, Italy	Temperate mediterranean	5512
	Paris, France	Temperate forest in Europe	11,561


European test scenes	Amsterdam, Netherlands	Temperate forest in Europe	9714
	London, England	Temperate forest in Europe	6711
	Munich, Germany	Temperate forest in Europe	7355


Test scenes beyond Europe	Beijing, China	Temperate forest in East Asia	11,017
	Nairobi, Kenya	Tropical, sub-tropical savannah in Africa	591
	Rome, Italy	Temperate mediterranean	2890
	Rio de Janeiro, Brazil	Tropical, Sub-tropical savannah in South America	2492
	San Francisco (SF), USA	Temperate mediterranean	1784
	Santiago, Chile	Temperate mediterranean	2890
	Sydney, Australia	Temperate forest in North America	1894
	Tehran, Iran	Temperate grassland in Middle East Asia	1678
	Jakarta, Indonesia	Tropical, Sub-tropical forest in Asia	2492
	New York City (NYC), USA	Temperate forest in North America	7355

**Fig. 5:**
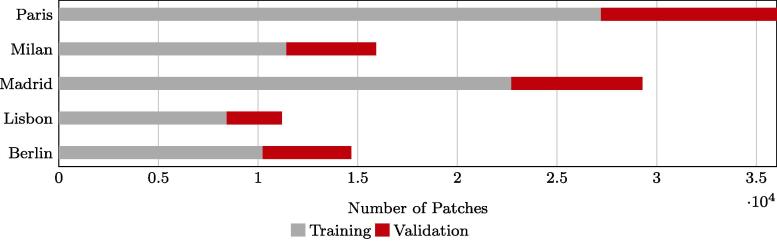
Number of training and validation patches in our dataset.

**Fig. 6:**
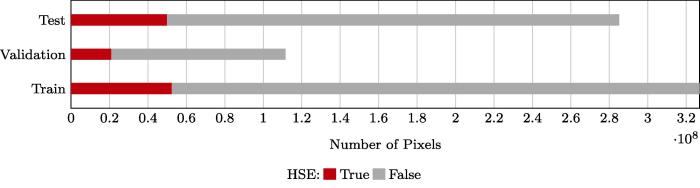
Number of pixels in training, validation, and test datasets. The test data presented here is from the three scenes in Europe.

After coregistration, HSE reference data and Sentinel-2 images were cropped into patches of 128 × 128 px with a stride of 96 px. The final patches were spatially split into a training and a validation subset. The exact number of patches from each training scene is presented in Fig. 5. The number of HSE and non-HSE pixels in the training, validation, and test datasets in Europe is presented in Fig. 6.

### 3.2 Accuracy assessment strategy

**Fig. 7:**
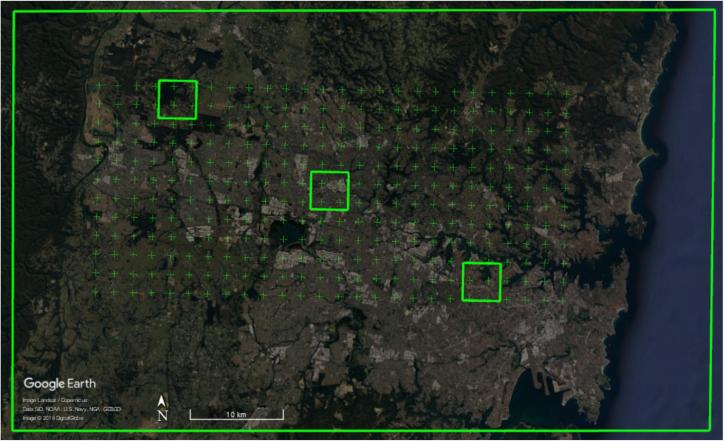
The MLGCPs and three subset regions for closer visualization within the ROI, using the city of Sydney as an example. A similar configuration is used for the assessment of all test cases.

**Fig. 8:**
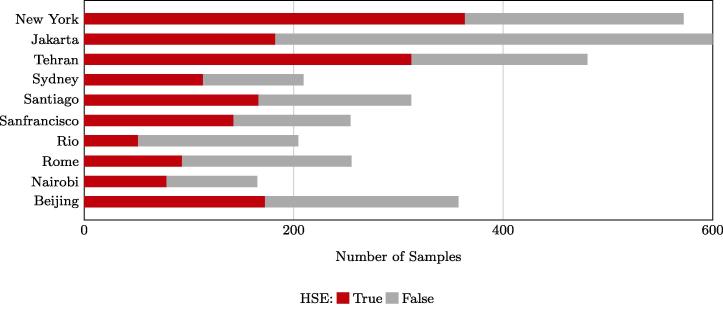
Number of MLGCPs for HSE mapping assessment. A different number of points are chosen for different cities to ensure diversity in land covers by including different city areas.

Manually labeled ground truth is employed for a quantitative assessment. In order to avoid human-induced bias, an equally distributed grid is generated for each test city, in the city center area, with 2000 m distance between each point. These manually labeled grid-based checking points (MLGCPs), with a size of 20m×20m, are manually classified into HSE or non-HSE. This fixed distribution of check points allows for a meaningful spatial assessment of the mapping results. For similar reasons, three fixed subset regions, with a size of 4km×4km, distributed across the whole region of interest (ROI), are chosen for each city for a closer view of the produced results. Fig. 7 illustrates the three subset regions and the MLGCPs within the ROI, using Sydney as an example. The number of test samples of all ten test scenes is presented in Fig. 8.

Furthermore, several state-of-the-art products were chosen for comparison based on the following riteria: they should be available on a global scale, be provided with a similar pixel spacing, and provide relevant information about HSE, because only similar characteristics enable an extensive and consistent comparison. Therefore, we chose GUF, the GHS built-up grid, FROMGLC10, and High-resolution Multi-temporal Global Urban Land (HMGUL) (Liu et al., 2018) as the baselines for comparison and validation of the HSE mapping results produced by our approach. The details of these reference products are provided in Table 2. All baseline products were re-sampled to 20 m GSD for comparison with the produced maps in this study. For the purpose of comparison, the “built-up” and “built-up up to 2014” are taken from GUF and GHSL as the HSE information, respectively. Because neither of these products should be considered as ground truth, as they were all created by different mapping approaches, we do not test our results against them. Instead, we compare our results to these datasets with respect to independent references.

**Table 2:** Description of baseline products for accuracy comparison.

Product	Sensor	Year	Label	GSD
GUF	TerraSAR/TanDEM-X	2011–2014	Built-up, with vertical component	12
GHSL	Landsat	1975–2014	Multi-epoch built-up grid	38
FROMGLC	Sentinel-2	2017	Impervious surface	10
HMGUL	Landsat	2015	urban	30

In addition to quantitative and visual comparisons with similar products, a quantitative assessment is also performed with respect to the OpenStreenMap building layer used as the ground truth reference. Because the mapped HSE includes not only buildings but also other man-made structures, such as roads, we only employed recall as the indicator. That is $\mathrm{recall}=\frac{N_{1}}{N_{0}}$, where $N_{0}$ is the number of all building pixels based on OSM, and $N_{1}$ is the number of pixels (in $N_{0}$) also mapped as HSE. This way, we are aiming at the detection rate of buildings in the mapping results. A good HSE map should include all buildings provided in the OSM building layer. It should be mentioned that the quality of the crowdsourced OSM reference data is not homogeneous over the cities and the suburban areas, as well as over developing and developed countries, in terms of completeness and thematic accuracy (Fan et al., 2014, Arsanjani et al., 2015, Johnson et al., 2017, Viana et al., 2019). Therefore, in our study, the OSM-based evaluation results are only provided as an additional rough accuracy estimate of the HSE mapping results and should be primarily used for a relative comparison of the results. Additionally, buildings are also included in both the GHS built-up grid and GUF datasets, according to their definitions. Therefore, the detection power of these two layers is also presented by the above defined recall metric for comparison, in order to gain an intuitive estimation of the quality of the mapped HSE.

## 4 HSE mapping results

The results of the experimental assessment of the proposed HSE mapping framework are illustrated in this section. First, accuracy assessments with respect to different reference data are shown. We then compile the comparison between the mapped HSE and the state-of-the-art products for several cities across the world. For better evaluation, we visualize the comparison at both the city scale and building block scale. Finally, case studies for large-scale HSE mapping are provided to demonstrate the upscaling potential of the proposed framework.

### 4.1 Quantitative assessment of HSE mapping results

For the ten globally distributed cities, accuracy assessments are carried out with two kinds of reference data, MLGCPs and OSM. The kappa coefficient, average accuracy (AA) of the two classes (HSE and non-HSE), commission error, recall, and F-Score of HSE are shown in Table 3. To provide a sense of the quality of the achieved results, we also list the corresponding assessment results for the state-of-the-art products, GUF and GHS.

**Table 3:** Accuracy assessment of HSE mapping results from Sen2HSE-Net by kappa, AA (in percentage), commission error (CME, in percentage), recall (in percentage), and F-Score with respect to the MLGCPs and OSM reference data. The corresponding assessment of GUF and the GHS built-up grid is also listed for comparison. Only recall with respect to OSM is presented, given the different definitions of HSE and OSM reference.

Reference		Source	Beijing	Nairobi	Rome	Rio	SF	Santiago	Sydney	Tehran	Jakarta	NYC	**Mean**
MLGCPs	Kappa	ours	**0.75**	**0.73**	**0.79**	**0.88**	**0.88**	**0.90**	0.78	0.67	**0.89**	**0.82**	**0.81**
		GUF	0.64	0.70	0.75	0.81	0.81	0.75	**0.81**	**0.76**	0.60	0.62	0.73
		GHSL	0.54	0.37	0.77	0.74	0.87	0.65	0.77	0.70	0.36	0.72	0.65

	AA	ours	**87.6**	**86.1**	**88.2**	**94.4**	93.8	**94.8**	88.6	81.8	**94.4**	**91.0**	**90.1**
		GUF	81.9	84.7	85.9	89.0	88.9	88.4	**91.0**	**88.5**	80.7	83.9	86.3
		GHSL	77.2	68.0	87.8	90.1	**95.4**	83.7	88.4	85.6	65.9	85.3	82.7

	CME	ours	17.4	4.8	6.3	9.6	**6.8**	5.9	15.8	15.1	**8.2**	6.6	9.7
		GUF	**16.3**	3.4	**5.5**	**5.8**	8.9	**3.4**	**4.6**	**6.0**	12.7	**4.3**	**7.1**
		GHSL	26.1	**3.3**	11.6	26.9	14.0	4.7	13.8	9.0	26.3	11.7	14.7
	recall	ours	**93.6**	**75.6**	79.6	92.2	**96.5**	**96.4**	**99.1**	**95.2**	92.3	**93.4**	**91.4**
		GUF	77.9	71.8	74.2	80.2	80.4	80.3	86.7	83.2	84.0	73.6	79.2
		GHSL	80.8	37.2	**81.7**	**95.6**	96.1	71.8	94.0	80.5	**94.2**	91.7	82.4
	F-Score	ours	**0.88**	**0.84**	**0.86**	**0.91**	**0.95**	**0.95**	**0.91**	**0.90**	**0.92**	**0.93**	**0.91**
GUF		0.81	0.82	0.83	0.87	0.85	0.88	**0.91**	0.88	0.86	0.83	0.85
GHSL		0.77	0.54	0.85	0.83	0.91	0.82	0.90	0.85	0.83	0.90	0.82


OSM	recall	ours	**97.9**	**92.2**	**93.8**	93.3	**99.1**	**99.1**	**97.8**	**97.6**	**98.1**	97.7	**96.7**
		GUF	89.7	84.2	90.1	84.1	77.6	91.2	87.0	87.8	81.5	90.1	86.3
		GHSL	92.9	72.6	92.1	**97.3**	98.0	72.2	96.9	73.0	96.4	**97.9**	88.9

The numbers in bold are the highest accuracy among the three layers.

Table 3 indicates that the achieved HSE mapping results are promising, as they provide the highest kappa, AA, recall, and F-Score on average over ten test scenes, when compared to both of the baseline products. In particular, we achieve the highest F-Score (with respect to the MLGCPs) for all ten distinct test areas across the world. In addition, more buildings (from the OSM layer) are included in the mapping results, compared to both GUF and the GHS built-up grid. This can be seen from the improved mean recall, from 86.3% and 88.9% to 96.7%, compared to GUF and the GHS built-up grid, respectively. This improvement is apparent for eight of the ten cities.

The commission error from our mapping results, however, is relatively high, especially when compared to GUF, which means that the HSE is overestimated in our results. On the one hand, this shows that GUF is strong at excluding non-HSE from HSE. On the other hand, it is also due to the different mapping focus (vertical artificial structures) of GUF. Still, even considering commission error, our results are generally better than the GHS built-up grid, which is closer to our mapping focus. The GHS built-up grid provides the highest recall in three test scenes with respect to the MLGCPs and two test scenes with respect to OSM. The differences among these three results will be further analyzed in the discussion section. Considering the varying characteristics of the three layers, it should be mentioned that the comparison presented in Table 3 is not intended to rank their quality, but rather to provide a validation reference for our mapping results through comparisons.

The presence of fewer outliers in the representative test scenes shows the good generalization ability and the robustness of the trained model. However, the achieved results do reveal differences among different test scenes. For instance, the result in Nairobi is worse than the average for all three dataset. This is probably due to different urban structures and surrounding terrains, and is indicative of the challenges for large-scale mapping.

### 4.2 Qualitative assessment of HSE mapping results

**Fig. 9:**
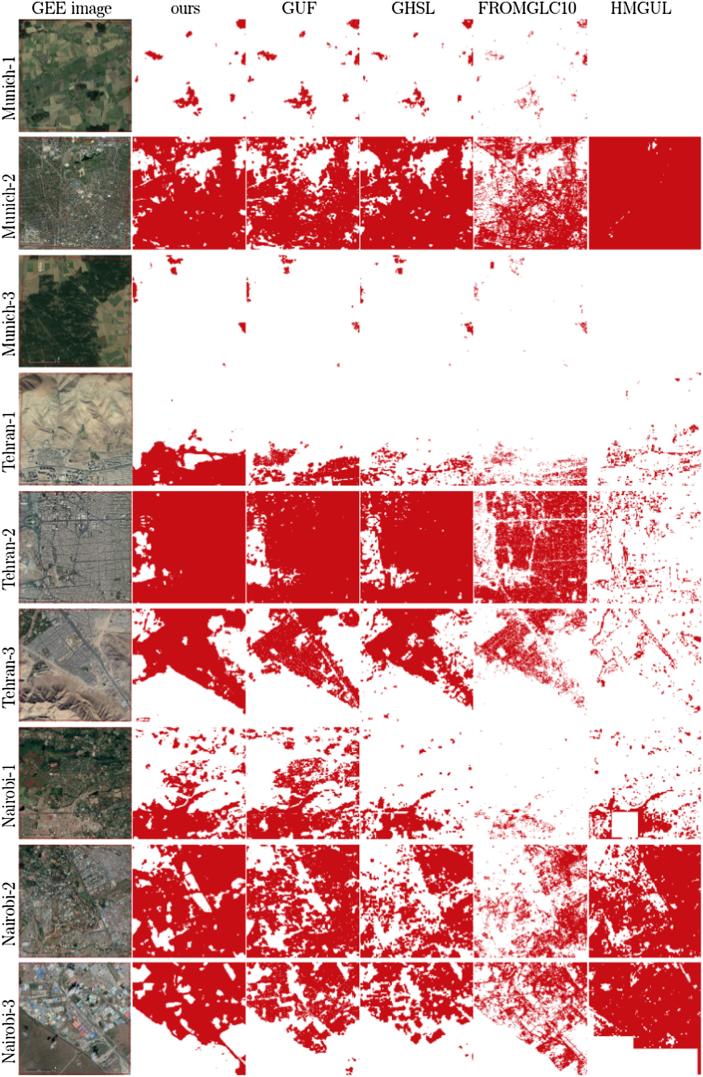
Comparison of the produced HSE results to four state-of-the-art products for three pre-defined subsets in three test scenes.

The comparison of the produced HSE maps to the state-of-the-art products can be found in Fig. 9 for the three subset test areas in Munich, Nairobi, and Tehran. Overall, the mapped HSE results are in agreement with the GHS built-up grid, GUF, and FROM-GLC10, while the HMGUL is in a relative coarse resolution. From the comparison, it can also be seen that the mapped HSE does include roads, streets, in addition to buildings, as expected. Some roads are also included in the GHS layer, FROM-GLC10, and HMGUL.

Some superiority of the mapping results can be observed from Fig. 9. For instance, the mapped HSE is able to exclude the park area within the city, as illustrated by the second Munich subset. Also, it is able to include small buildings surrounded by vegetation as well as GUF does, while the GHS built-up grid and FROM-GLC10 omit most of the buildings, as illustrated by the first Nairobi subset and the first Munich subset. Additionally, the proposed approach is not affected by the shadow areas of the mountains, which result in false positive results in the GHS built-up grid, as can be seen in the third Tehran subset.

**Fig. 10:**
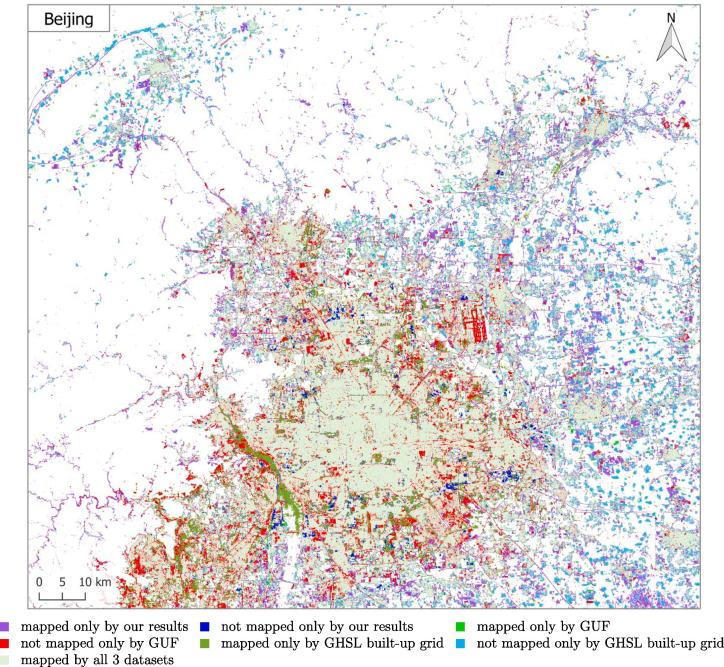

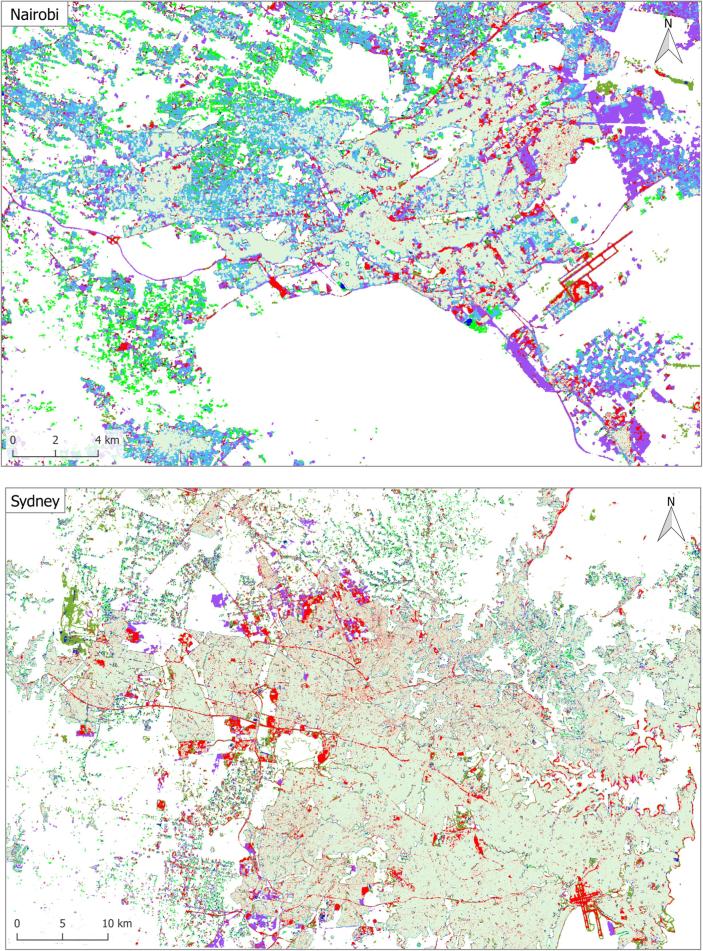
Produced HSE maps of three representative test scenes, compared to the reference GUF and GHSL built-up grid datasets.

**Fig. 11:**
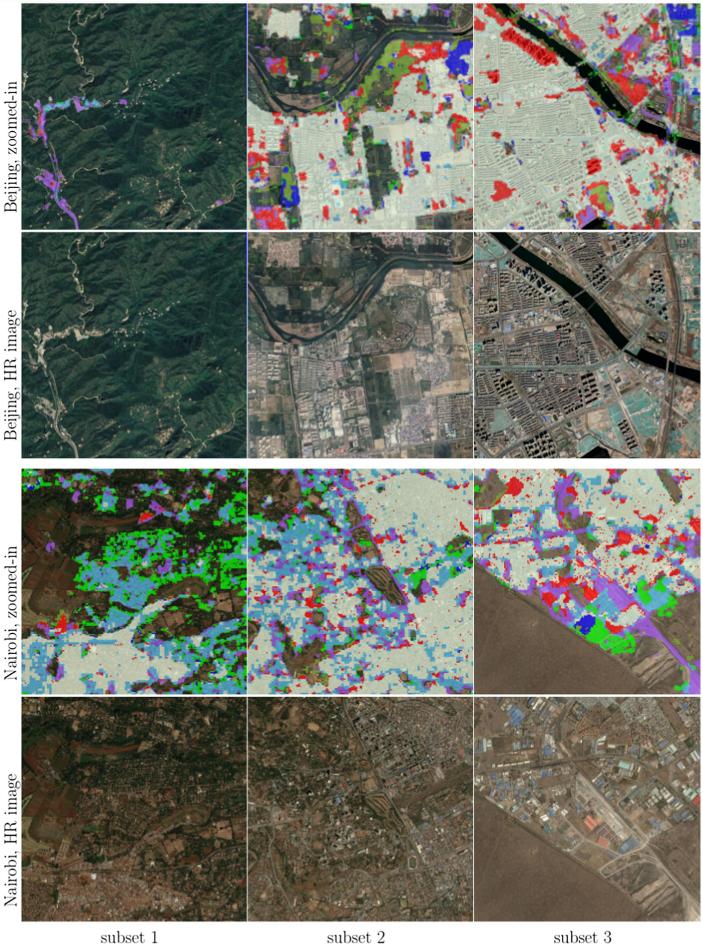

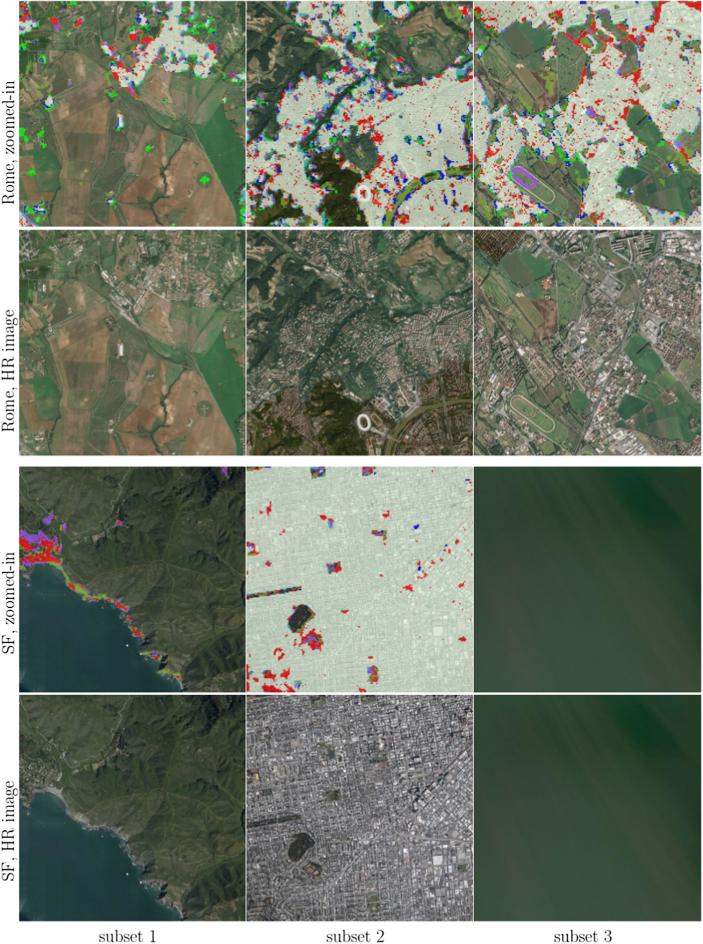

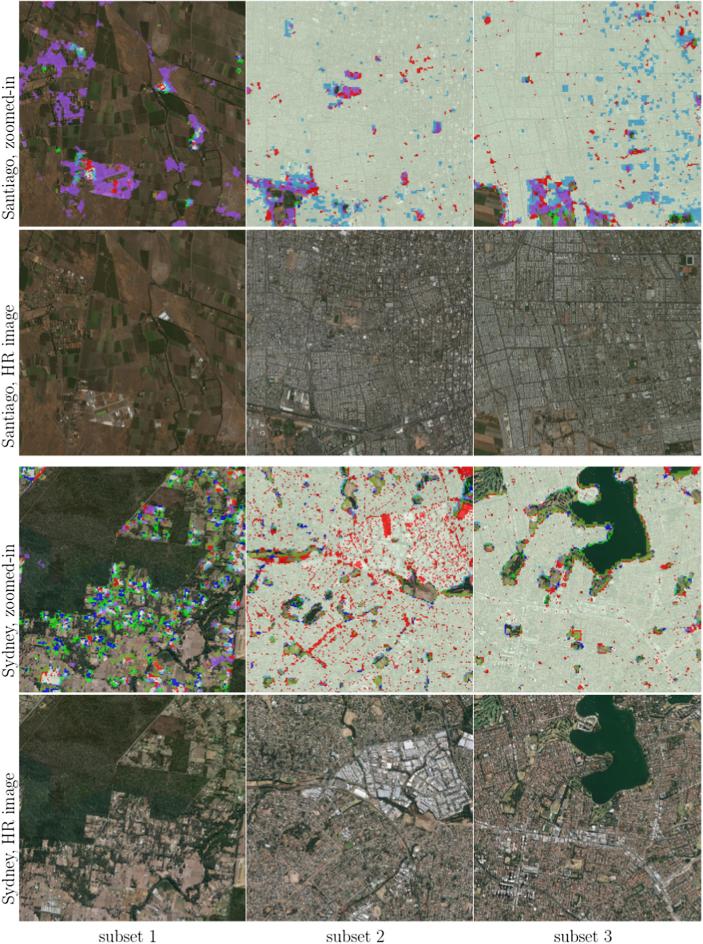
Closer view of the three subsets of sample test scenes distributed across the world, overlaid on high resolution images. The high resolution images are also shown for detailed interpretation.

For a city-scale evaluation of the mapped HSE, the similarities and differences from the GHS built-up grid and GUF are shown in Fig. 10 for three representative test scenes. The visualization can be interpreted using Table 4. The closer view of the three pre-defined subset regions (as described in Section 3) of six sample test scenes in Beijing, Nairobi, Rome, San Francisco, Santiago, and Sydney, are shown in Fig. 11, where high resolution images are also presented for a detailed interpretation.

**Table 4:** Interpretation of the colors in Fig. 10. FP and FN are false positive and false negative, i.e., commission error and omission error, respectively.

Fig. 10 visualizes the overall consistency and agreement of the produced HSE with respect to the GHS built-up grid and GUF. From the test cases in Beijing and Sydney shown in Fig. 10, it can be seen that the main part of a city can be detected by all three datasets, with the urban morphology being shown clearly. The test in Nairobi shows obvious disagreement among these three datasets, which is also noticeable in the other test scenes and will be further analyzed in the discussion section. Fig. 10 qualitatively shows the general feasibility of the proposed HSE mapping framework and can be further confirmed by the closer view in Fig. 11. By comparing the high resolution images in Fig. 11, we can see that in general our results are able to provide a compact boundary between HSE and non-HSE under a variety of environments in cities across the world. A detailed analysis of this visualization will be presented in the discussion section, providing more evidence of the outstanding performance of the proposed framework.

### 4.3 Examples of regional-scale and country-wide HSE mapping

**Fig. 12:**
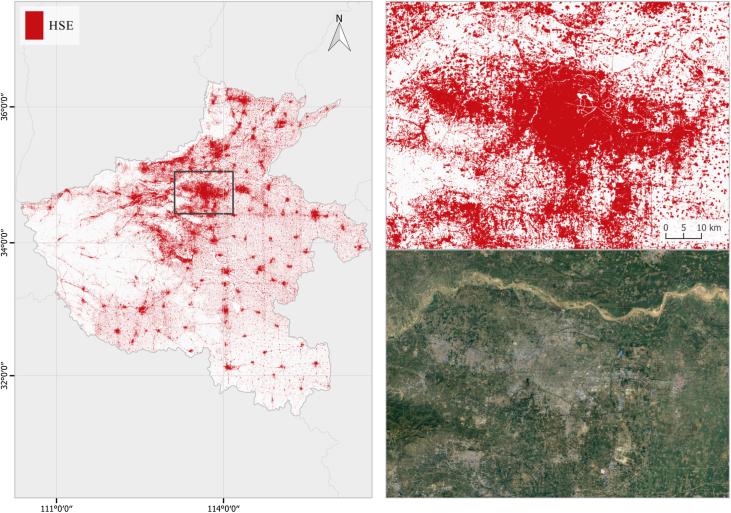
Regional HSE mapping example in Henan (province), China. The Zhengzhou (city) area is zoomed in and compared to a high resolution image.

**Fig. 13:**
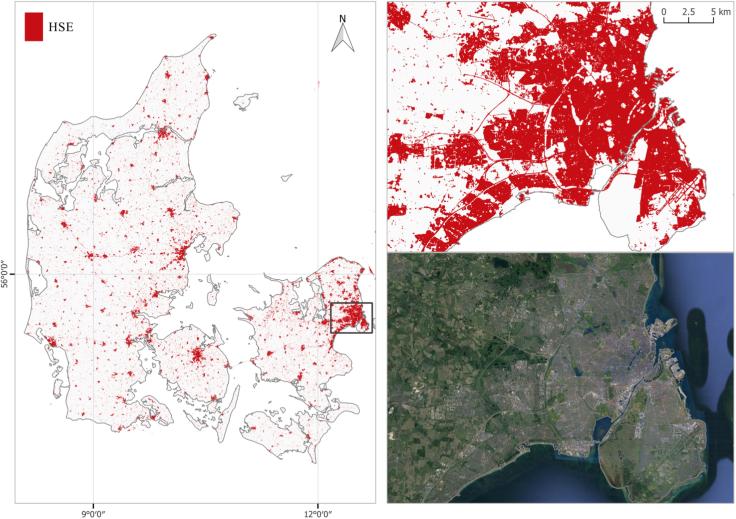
Country-wide HSE mapping example in Denmark. The Copenhagen area is zoomed in and compared to a high resolution image.

In order to validate the stability of the proposed framework, we tested the workflow on a regional-scale and country-wide HSE mapping task, in Henan province, China and in Denmark. The total area of each is about 167,000 and 42,933 $km^{2}$, respectively. The HSE mapping results are shown in Fig. 12, Fig. 13. The general urban pattern is successfully mapped for both examples, as can be seen when they are compared with high resolutions satellite images. This test demonstrates the general performance and the potential for upscaling of the presented framework.

## 5 Discussion

In the section, we provide some empirical evidence of the framework setup and network design, as well as addressing the problems and questions posed in Section 1, using insights gained from the extensive experimental results presented in Section 4 and some additional investigations. This section will also discuss some lessons learned that are relevant to similar topics and further possible improvements toward more accurate and operational HSE mapping.

### 5.1 Choice of the proposed framework

To demonstrate the rationale behind our design choice, this section provides some sensitivity analyses. The achieved results are first compared to those from several state-of-the-art baseline methods in Section 5.1.1. In addition, two different ways of splitting of training and validation data are compared to justify our experimental setup. Last, the effect of network depth and width are investigated for the employed architecture, to provide more insights into our approach.

#### 5.1.1 Comparison with baseline methods

The achieved HSE mapping results from the proposed Sen2HSE-Net are compared to those from baseline networks in Table 5 for test, both beyond and within Europe. Table 5 shows that the proposed shallow network with 9 layers is able to provide even better mapping accuracy than the much deeper and relatively complicated U-Net (Ronneberger et al., 2015), with more trainable parameters. In addition, the achieved results from Sen2HSE-Net are much more accurate than those from ResNet-PSPNet (Zhao et al., 2017), ResNet-FCN-8 (Long et al., 2015), and attention-based FCN (Fu et al., 2018), which have been shown to be more powerful for detailed semantic segmentation. One possible reason is the information loss from the pooling layers in the encoding process (by ResNet), which is not suitable for our HSE mapping task and Sentinel-2 data. Furthermore, this loss cannot be compensated, even with the sophisticated design of the decoding part, either with pyramid scene parsing by ResNet-PSPNet, or upsampling with low-level features considered by ResNet-FCN-8, or attention modules proposed in (Fu et al., 2018). These observations confirm the assumptions that motivate our framework design: good performance is not guaranteed when simply and directly using the state-of-the-art networks for remote sensing tasks. Instead, characteristics of both the task and data need to be integrated into the network design. Additionally, Table 5 shows that it is possible to use a simple FCN to achieve promising HSE mapping results, instead of relying on the existing rather sophisticated networks. Even though comparable results can be achieved from directing employing U-Net, the proposed Sen2HSE-Net is much lighter, which is significant for large-scale mapping.

**Table 5:** Results from Sen2HSE-Net and three baseline semantic segmentation networks, tested in areas beyond and within Europe.

Method	test beyond Europe					test in Europe					network	
	Kappa	AA	recall	F1		Kappa	AA	recall	F1		layer	# of Para.
Sen2HSE-Net	0.809	90.1%	91.4%	0.906		0.802	90.5%	84.4%	0.834		9	1,124,866
U-Net (Ronneberger et al., 2015)	0.804	90.3%	90.4%	0.903		0.788	89.3%	81.7%	0.822		24	31,036,872
ResNet-PSPNet (Zhao et al., 2017)	0.655	82.8%	80.3%	0.824		0.644	80.3%	64.5%	0.697		58	28,550,594
ResNet-FCN-8 (Long et al., 2015)	0.740	87.2%	89.3%	0.875		0.719	84.8%	73.0%	0.762		60	31,960,710
FCN + dual attention (Fu et al., 2018)	0.785	89.4%	86.3%	0.888		0.760	85.2%	72.4%	0.795		27	14,405,056

#### 5.1.2 Effect of training and validation data split

Testing performance depends on how the training and validation datasets are split, because validation data provides hints of the progress during training and is the basis for choosing the best trained model. To understand the influence of the choice of validation data on the eventual test results, we have investigated two different variants of validation data selection. It has to be noted that the validation data is always chosen as a subset of the training set from the training scenes, whereas the test data in this study always came from test scenes and remained unseen during training.

This effect is shown in Table 6, with both the proposed Sen2HSE-Net and the standard segmentation network, U-Net, as examples. Random split is randomly choosing about 25% of the data from each training city as the validation dataset, while spatial split is extracting about 25% of the upper left part of each city as the validation dataset. Spatial split is also what is used in this study. As described in Table 1, the models are tested on test data that are completely unseen during training. From the illustration in Table 6, we can see that spatial split is better than random split, since almost all metrics are better from a spatial split, which is true for both networks. This may be because the distribution of training and validation data is more similar in random split than spatial split, which leads to a validation accuracy that is closer to the training accuracy. As a result, the chosen model is optimal for the training areas, rather than the unseen test areas.

**Table 6:** Results from different approaches to splitting of training and validation datasets, tested in completely unseen areas both beyond and within Europe.

Network and data split		Test beyond Europe					Test in Europe			
		Kappa	AA	recall	F1		Kappa	AA	recall	F1
Sen2HSE-Net	spatial	0.809	90.1%	91.4%	0.906		0.802	90.5%	84.4%	0.834
	random	0.788	89.1%	87.7%	0.891		0.798	89.6%	82.1%	0.830
U-Net	spatial	0.806	90.0%	90.2%	0.902		0.801	89.2%	81.1%	0.832
	random	0.805	90.1%	87.7%	0.897		0.791	88.5%	79.5%	0.824

#### 5.1.3 Effect of network depth and width

It is important to know whether better HSE mapping results can be achieved from a deeper and wider version of Sen2HSE-Net using the setup of this study. Table 7 sheds light on this potential improvement, by comparing the results of one wider and three deeper versions, as well as the number of trainable parameters in each network. From these comparisons, we observe no gain from a wider network and a slight improvement from a deeper network. Interestingly, the improvement is not present when the depth increases further, from depth 13 to 17 and 21. This might result from the characteristics of the task, the use of Sentinel-2 images, which are not high resolution, as well as the testing choice (in unseen areas).

**Table 7:** Results from Sen2HSE-Net of varying depth and width. All comparing networks employ the same overall architecture as Fig. 3. The result in the first row is from the configuration used in Section 4.

Network				test beyond Europe					test in Europe			
# of first Conv	layer	# of Para.		Kappa	AA	recall	F1		Kappa	AA	recall	F1
f = 16	2 + 2 + 2 + 2 + 1	1,124,866		0.81	90.1%	91.4%	0.91		0.80	90.5%	84.4%	0.83
	3 + 3 + 3 + 3 + 1	1,874,098		0.82	90.6%	93.1%	0.91		0.80	90.4%	84.2%	0.83
	4 + 4 + 4 + 4 + 1	2,623,330		0.81	90.1%	91.5%	0.90		0.80	90.7%	85.0%	0.84
	5 + 5 + 5 + 5 + 1	3,372,562		0.80	89.7%	92.5%	0.90		0.80	90.3%	84.2%	0.83
f = 32	2 + 2 + 2 + 2 + 1	4,493,826		0.81	90.1%	90.0%	0.90		0.80	89.0%	80.7%	0.83

### 5.2 Analysis of the HSE mapping framework

While the quantitative and qualitative results presented in Section 4 have shown the promising performance of our framework, there are some details requiring analysis for a better understanding of both the method and the produced results. These details will be addressed in this subsection.

#### 5.2.1 Mapping power of the proposed framework

**Fig. 14:**
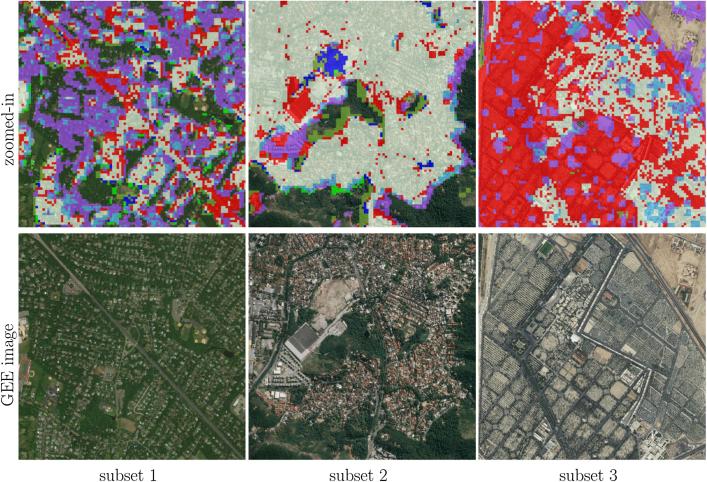
Closer view of some positive examples, with the same legend as in Fig. 10. Colors can be interpreted according to Table 4.

The goal of this study is to explore a better solution for mapping HSE with the potential of upscaling. Fig. 14 illustrates the HSE mapping power, with some positive examples in test scenes in New York City, Rio, and Tehran. In the examples in Fig. 14, only our solution is able to include sparse buildings and buildings on the boundaries, surrounded by trees and gardens, as the purple outlines indicate the areas that are only mapped by our results and are missed by the other two baseline products. This can also be observed in the first subset in Beijing, the first subset in Nairobi, and the first subset in Santiago, as shown in Fig. 11. In the second subset of Fig. 14, only our result is able to exclude the soil ground from the mapping results, as the blue outlines indicate areas mapped by other layers but not by our results. This can also be seen in the second subset of Beijing and the third subset of Nairobi, as shown in Fig. 11. The red and cyan color outlines indicate areas that are not mapped by GUF and GHSL, respectively. Since these areas are mapped not only by our results but also by one of the baseline datasets, they are very likely HSE, and correctly detected by our approach. This can be seen from the third subset in Fig. 14, as well as the first subset in Nairobi, the first subset in Rome, and the second subset in Sydney in Fig. 11.

**Fig. 15:**
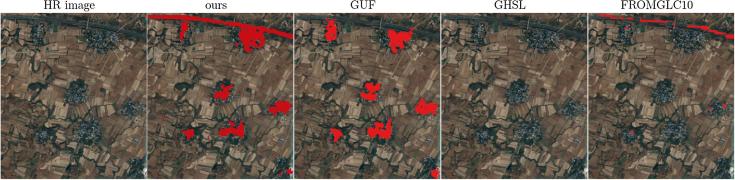
Closer view of the HSE mapping power of the proposed framework, with an example around the location of longitude 113.2072 and latitude 32.6849.

More evidence of the mapping power of the proposed framework can be seen in Fig. 15, a close-up of Fig. 12, where we are able to detect buildings in small villages as well as GUF, which is derived from very high resolution SAR images. The other products unfortunately fail to map these areas. This also shows the improvement of space over current land cover mapping at global scale, especially in rural areas.

Jointly considering the accuracy assessment with respect to the MLGCPs and the OSM building layer shown in Table 3, as well as the visualizations at different scales in Fig. 10, Fig. 11, we conclude that HSE maps can be created by the proposed approach, with comparable or even better quality than state-of-the-art products. Generally good results can be achieved, even in test cities with various typologies of urban areas and vegetation, different climate, and diverse culture regions. This finding suggests the proposed framework's potential for generalizing and upscaling. Furthermore, the assessment of the experimental results provides evidence that the motivation for setting up the framework is valid. That is simple FCNs and the multi-spectral images from the Sentinel-2 mission are indeed valuable for large-scale HSE mapping and could be exploited to produce large-scale HSE maps with a 20 m GSD. Also, this work demonstrates that not having highly accurate pixel-level ground truth does not hinder the successful adaptation of deep neural networks to the application of HSE mapping.

**Fig. 16:**
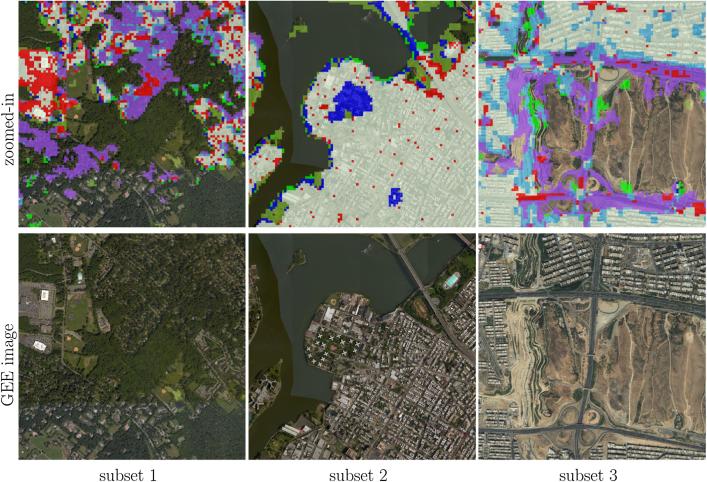
Closer view of some negative examples, with the same legend as in Fig. 10. Colors can be interpreted according to Table 4.

However, some problems in the current mapping results remain, as shown by the negative examples from test scenes in New York City and Tehran in Fig. 16. In the first subset, there are still some buildings omitted by our mapping results, and in the second subset, there is still an area omitted only by our approach. In addition, some overestimation can be seen in the third subset; this can also be observed in the first subset of Rome and the third subset of Santiago in Fig. 11. This overestimation, i.e., a commission error, is inherent to the definition of the task and the setup of the framework. Specifically, the goal is to detect whether there is HSE in a 20 by 20 m cell. Therefore, the boundaries tend to be identified as HSE. Possible approaches for improvement will be proposed in Section 5.3.

#### 5.2.2 Differences between HSE mapping results and baseline products

**Fig. 17:**
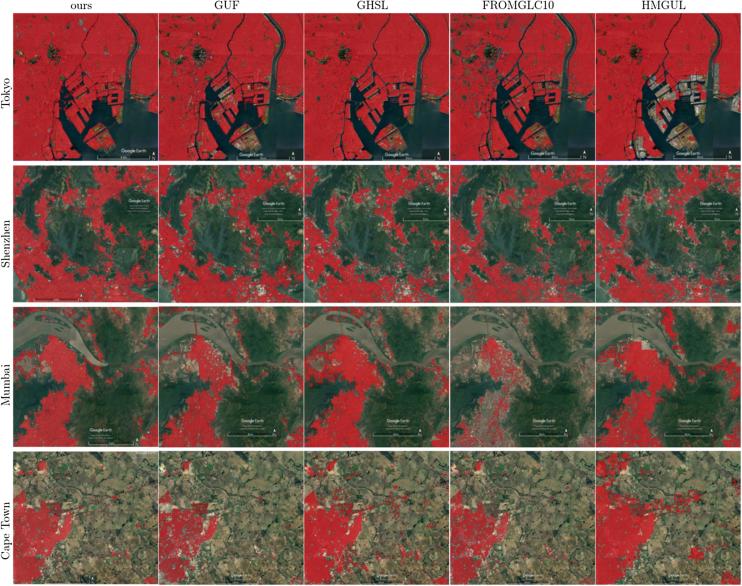
Differences among HSE-related datasets. Red areas are mapped areas from existing products based on Table 2. Four distinct areas are chosen to present highly heterogeneous urban structures from different parts of the world.

Comparisons in Section 4 also reveal some notable differences among our HSE mapping results, GHS built-up grid, GUF, FROM-GLC10, and HMGUL. These differences are further visualized in Fig. 17 for four distinct areas around the world. Similar to the HSE mapped by our approach, both GHSL and HMGUL include not only buildings but also impervious surfaces such as roads and parking lots, even though they are not focused on impervious surfaces. This is because the medium-resolution data employed is not enough to exclude small gaps among buildings, especially when the gaps are covered by the same materials as buildings. It is thus challenging to distinguish these areas that are highly related to HSE and bear a similar spectral signature as buildings when using the spectral information from optical satellite images. In contrast, GUF does not contain such impervious surfaces, as can be seen from the red regions in Fig. 10, Fig. 11. This is because GUF focuses more on vertical building structures, removing roads and paved surfaces during the post-editing period (Esch et al., 2017). It is also due to the peculiarities of the SAR images used for the production of GUF. The local speckle information and the texture information in the SAR images makes it possible to specifically detect vertical structures such as buildings (Klotz et al., 2016, Qiu et al., 2018). Specifically, buildings are characterized by stronger back-scattering signals than airport roads, even though they are made of the same materials. However, when using optical satellite images, it is challenging to distinguish different land covers within the super-class of impervious surfaces, as they share similar spectral signatures. An illustrative example is the Sydney Airport (the red cross-shape in the lower right corner of Fig. 10), where the aircraft runways are mapped as built-up areas in both GHSL and the result of this study.

Also due to the peculiarities of the SAR images used for the production of GUF, some sparse trees can be mistaken as buildings, as shown in the the first and third subset of Nairobi in Fig. 11. For GHSL, error prone areas are forests and bodies of water, as shown in the second subset of Rome, the first subset of San Francisco, and the third subset of Sydney. As a result of the two phenomenon discussed above, sparsely built-up areas surrounded by sparse forest can be challenging, as can be seen from the “noisy” visualizations in the suburban areas in Fig. 10.

In Fig. 17, it can be seen that FROM-GLC10 and HMGUL are subject to obvious omission errors in Mumbai and Tokyo, respectively, providing one more piece of evidence for the proposed approach's improved performance over state-of-the-art layers. A further comparison between our results and GHSL shows that more roads are mapped by our approach, as shown by the purple lines in the Nairobi and Beijing test scenes in Fig. 10, demonstrating the powerful mapping capability of our framework.

These characteristics of each product discussed above relate to the differing definitions of “urban,” “human settlement,” and “built-up,” as well as the the mapping approaches employed and the datasets used. End users of these products in particular should take note of these differences. On the other hand, understanding these differing characteristics also makes it possible to extract complementary information from different products for various applications.

It should be mentioned that all comparisons in this study are intended merely to provide an assessment with reference to the state-of-the-art products. The occasional inferior performance of the GHS built-up grid and GUF is certainly partially due to temporal gaps in data collection: the ongoing urbanization of the world has changed many originally suburban areas to newly built-up areas after the GHS built-up grid and GUF were released. This cannot be easily ignored, especially for cities in developing countries, such as Beijing. This issue highlights the necessity for up-to-date worldwide HSE information, in addition to the existing products: GUF, with its unprecedented spatial resolutions, the GHS built-up grid, with its multi-temporal resolution, and FROM-GLC10, with its detailed land cover information.

### 5.3 Further improvements toward operational mapping

We are able to achieve state-of-the-art HSE results for several representative scenes across the world. Furthermore, comparable accuracy is achieved for both regional mapping (three test scenes in Europe) and large-scale mapping (the ten world-wide distributed test cities), as shown in Table 5. However, there is still much room for further improvements toward an operational large-scale—even global—process. The improvements can mainly be achieved with respect to three aspects: the input satellite images, the deep neural network architectures, and the post-processing of the mapped HSE results. First, Level-2A Sentinel-2 images (bottom-of-atmosphere reflectance) and the spectral ratios could bring accuracy improvement. In order to produce HSE maps at a regular frequency, it is not enough using Sentinel-2 images alone, especially in regions with heavy cloud cover throughout the year such as the Southeast Asia (Stengel et al., 2017). One solution is to employ multi-sensor, multi-temporal, and multi-modal data fusion, thus improving accuracy and enhancing temporal and spatial sampling (Schmitt and Zhu, 2016, Ghamisi et al., 1812, Lefebvre et al., 2016, Hong et al., 2019a, Hong et al., 2019b, Hong et al., 2019c, Qiu et al., 2019). Considering the scale and aiming applications, Landsat-8 and Sentinel-1 images could also be exploited for HSE mapping. It should be mentioned that the proposed framework can be easily adapted for these two datasets after proper preprocessing, like filtering for SAR images and cloud removal for optical images. In addition to the input images, improvement can also be realized via an ensemble with other deep CNNs in order to take advantage of their complementary characteristics and heterogeneous properties, as demonstrated by (Noh et al., 2015). Furthermore, the performance of the proposed framework should be further investigated and evaluated in rural areas, where built-up areas tend to be sparse and can be easily omitted. Finally, once the HSE results are acquired, further post-processing could be carried out independently for each city. For instance, a conditional random field could be applied to the output mapping results, in order to homogenize the segmentation (Maggiolo et al., 2018). Furthermore, in this process, any locally available datasets such as census data, as well as prior knowledge, could be exploited. Other directions worth exploring include adapting the trained model with semi-supervised learning-based strategies and transfer learning, including multitask learning, domain generalization, and domain adaptation, for the purpose of better generalization (Tuia et al., 2016).

## 6 Conclusions and outlook

Detailed and up-to-date HSE maps provide essential information about the human footprint on the earth, thus making sustainable development possible via proactive conservation. This paper presents a framework for large-scale HSE mapping from Sentinel-2 images, by exploiting a shallow yet effective FCN for semantic segmentation. In particular, the newly proposed framework takes advantages of globally available images from the Sentinel-2 mission, featuring medium spatial resolution, high revisit time, and multi-spectral imaging. As demonstrated in this paper, higher accuracy than state-of-the-art products can be achieved with the proposed approach. Our main conclusions and contributions can be summarized as follows:

• We propose a deep learning-based framework for large-scale HSE mapping from medium resolution Sentinel-2 images (10 m and 20 m GSD) with a small amount of reference data (with a temporal gap) from Europe. No manually labeled data is needed in the framework. This framework is potentially applicable for images from other satellites, such as Landsat and Sentinel-1, and the specific network architecture used in this study can be replaced by other state-of-the-art architectures or improved versions.
• We propose the use of a simple FCN instead of the sophisticated ones originally proposed for high resolution images, to avoid overhead and facilitate upscaling. The design choice of the framework is supported by comparisons with several baselines and investigations on the depth and width of the network as well as the experimental setup.
• We achieve HSE mapping results that are better than the state-of-the-art products, for several representative cities from six continents across the world. In order to carry out a fair comparison among different products and avoid human behavior-induced bias, two approaches for quantitative assessments, in addition to city-scale and building block-scale visualizations, are performed. Differences among HSE-related datasets are analyzed. HSE mapping examples at regional and country scale demonstrate the general performance of the framework.

We hope that our work encourages the explorations of the deep-learning-based approaches along with the rich array of geo-coded products for large-scale urban mapping. To this end, we will publish the trained models so that researchers can extract the HSE information of a specific region of interest via the proposed framework. Trained models and sample data are available at https://github.com/ChunpingQiu/Human-settlement-extent-detection-from-Sentinel-2-images-via-fully-convolutional-neural-networks-. Our future work includes further improving the mapping results of a specific region of interest. Additionally, the newly acquired Sentinel-2 images will allow for more timely and frequent HSE mapping and the 10- and 20-meter pixel spacing of Sentinel-2 images will allow for more detailed and accurate HSE mapping than those employing multi-spectral Landsat images with 30-meter pixel spacing. The promising results also motivate us to map more detailed multi-temporal HSE information from Sentinel-2 images in future.

## Declaration of Competing Interest

The authors declare that they have no known competing financial interests or personal relationships that could have appeared to influence the work reported in this paper.
